# Reduced Lung Function among Workers in Primary Coffee Processing Factories in Ethiopia: A Cross Sectional Study

**DOI:** 10.3390/ijerph15112415

**Published:** 2018-10-31

**Authors:** Samson Wakuma Abaya, Magne Bråtveit, Wakgari Deressa, Abera Kumie, Bente E. Moen

**Affiliations:** 1 Department of Preventive Medicine, School of Public Health, Addis Ababa University, P.O. Box 90861000 Addis Ababa, Ethiopia; deressaw@gmail.com (W.D.); aberakumie2@yahoo.com (A.K.); 2 Occupational and Environmental Medicine, Department of Global Public Health and Primary Care, University of Bergen, 5020 Bergen, Norway; Magne.Bratveit@uib.no (M.B.); Bente.Moen@uib.no (B.E.M.); 3 Centre for International Health, Department of Global Public Health and Primary Care, University of Bergen, 5009 Bergen, Norway

**Keywords:** coffee workers, dust exposure, Ethiopia, lung function, respiratory symptoms

## Abstract

Dust exposure is one of the major risk factors for respiratory health in many workplaces, including coffee factories. The aim of this study was to assess the prevalence of respiratory symptoms and lung function reduction among workers in Ethiopian primary coffee processing factories, compared to a control group of workers. A total of 115 coffee workers and 110 water bottling workers were involved in this study, from 12 coffee and 3 water bottling factories in Ethiopia, respectively. The chronic respiratory symptoms were assessed using a structured interview, using a standardized questionnaire adopted from the American Thoracic Society (ATS). The lung function tests were performed according to the ATS recommendation for spirometry. The coffee workers had a significantly higher prevalence of coughing, coughing with sputum, breathlessness, work-related shortness of breath, and wheezing compared with the controls. The prevalence ratio of work-related shortness of breath (PR = 3.7, 95% CI: 1.6–8.7) and wheezing (PR = 3.3, 95% CI: 1.3–8.4) was significantly higher for the coffee workers compared to the controls. The coffee workers in the age groups 28–39 years and ≥40 years, had a significantly lower forced vital capacity and forced expiratory volume in 1 s compared to the controls in the similar age groups. The findings indicated the need for longitudinal studies on the possible effect of coffee dust on respiratory health of coffee production workers.

## 1. Introduction

Several studies have indicated an association between working in coffee factories and respiratory health problems. Allergies have been suggested to be related to the problems [1,2,3,4,5,6,7]. A few older studies conducted in primary coffee processing factories have indicated that exposure to coffee dust is likely to cause acute and chronic respiratory symptoms in these factories as well [8,9,10,11]. 

Recently, studies were conducted in primary coffee processing factories in Tanzania. These demonstrated a higher prevalence of chronic respiratory symptoms in coffee workers than among the controls [11,12]. The primary coffee processing factories are factories that perform mechanical cleaning of the debris from the coffee-making process, such as the hulling, grading, hand picking, and packing of green coffee beans. The lung function parameters were not different between the coffee workers and the controls [12]. The Robusta coffee workers had higher prevalence of asthma symptoms than Arabica coffee workers [12]. The findings related to lung function were not clear, and more studies are needed for conclusive information regarding the respiratory health of coffee workers. Ethiopia produces exclusively Arabica coffee. The respiratory health impact of working with these coffee beans has not yet been explored. In addition, the coffee pre-processing methods used on the farms in Ethiopia is different from the ones used in Tanzania. Moreover, a recent study conducted in the coffee factories of Ethiopia found much higher levels of personal dust exposure compared with the levels measured in the Tanzanian factories [13].

In Ethiopia, more than 50% of foreign income comes from coffee, with an estimated 15 million people relying on coffee production for their livelihood [14]. Understanding the level of respiratory problems in Ethiopian coffee processing factories can generate information that could aid policy makers and other relevant stakeholders to develop any necessary preventive and control methods.

Studying respiratory disease is difficult in these types of factories, as a healthy worker effect may occur. This means that workers developing an illness may stop working, and may therefore not be found at the work sites. Cohort studies are difficult to perform, as the methods for tracing persons over longer periods is challenging. Case-control studies are also difficult to perform in Ethiopia, as the population does not have easy access to hospitals, and are treated at a large number of small health units. Therefore, a comparative cross-sectional study design was chosen, where the coffee factory workers were compared to a control group.

The aim of this study was to assess the prevalence of respiratory symptoms and to study the lung function among workers in the primary coffee processing factories of Ethiopia, and to compare these findings with a control group of water bottling workers. The methodology can detect early signs of respiratory diseases and is therefore useful in a company setting of relatively healthy workers.

## 2. Materials and Methods

### 2.1. Study Site and Period

There are about 746 primary coffee processing factories in Ethiopia. Almost all are found in three regions, Oromia; Addis Ababa; and Southern Nations, Nationalities and Peoples’ Region (SNNPR). We did a power calculation before beginning the study in order to estimate the required number of workers. Taking into consideration the available resources, we selected 12 primary coffee processing factories for inclusion in this study. The 12 primary coffee processing factories were divided equally among the three regions (i.e., four factories from each of the three regions), and the factories were selected randomly. The study was conducted from May to October 2016.

### 2.2. Control Group

The workers from three water bottling factories, one from each of the three study areas, were selected as the control group. Water bottling factories were chosen as their workers experience less dust exposure at work. Water bottling workers’ tasks are loading bottled water to the trucks. There are about 38 water bottling factories in Ethiopia.

### 2.3. Dust Exposure Levels

The personal total dust was sampled in the workers’ breathing zone using 25-mm three pieces, conductive cassettes with a cellulose acetate filter attached to a Side Kick Casella (SKC) pump with a flow rate of 2 l min^−1^ [15] Altogether, 360 full-shift exposure measurements were conducted on randomly chosen days of the week, and repeated sampling was conducted the next day. The sampling process is described in a previous paper [13]. A total of 60 full-shift personal exposure measurements were conducted in the water bottling factories. The arithmetic mean (range) of the personal total dust exposure was 17.36 mg/m^3^ (1.12–81.61 mg/m^3^) and 0.33 mg/m^3^ (0.11–1.16 mg/m^3^) for the coffee workers and controls, respectively. The personal total dust exposure levels among the coffee workers were significantly higher than among the control workers with geometric means (GM) of 12.30 and 0.30 mg/m^3^, respectively. The dust samples were analyzed gravimetrically using a standard microbalance scale AT261 Mettler Toledo with a detection limit of 0.01 mg/m^3^, in the accredited laboratory SINTEF MOLAB in Norway.

### 2.4. Study Population and Sample Size

The sample size for this study was calculated using a double population formula considering that the prevalence of wheezing among the primary coffee workers in Tanzania was 16% and 4.3% among the controls [12]. An 80% power was set to detect a difference in the wheezing between the two groups at significance level of 0.05. After considering 15% for non-response, a total of 240 participants (i.e., 120 from coffee factories and 120 from water bottling) were selected by systematic random sampling method, using the workers’ registration list as a sampling frame. All of the participants were male. In the coffee factories, only workers directly related to coffee processing were involved in this study. Office workers and guards were not included in this study.

### 2.5. Data Collection

#### 2.5.1. Chronic Respiratory Symptoms Interview

The chronic respiratory symptoms among participants were assessed with an interview, using a standardized questionnaire from the American Thoracic Society (ATS) [16]. This questionnaire was chosen because it has been used in previous occupational studies of respiratory health in East-Africa [12], making comparisons possible. The questionnaire was translated from English to Amharic and Afan Oromo languages, and translated back to English. A pre-test was conducted prior to the actual data collection for the validation of the data collection tool. Questions that were not easy for the participants to understand were rephrased to make them more easily understood. The interviews were conducted in an office located at their workplaces in absence of other people, in order to help the interviewee speak freely. The interviews lasted between 25 and 40 min per respondent.

The data were collected by the principal investigator together with an experienced research assistant. The questionnaire included socio-demographic data (age, height, weight, and Body Mass Index (BMI)), occupational history (years of work experience in the present and other dusty factories), past respiratory diseases (pneumonia, tuberculosis, bronchitis, asthma, and chest injury), and smoking habits (current smoker, ex-smoker, and never smoker). The questionnaire also included questions about the use of respiratory protective devices while working (yes/no), and the reason for not using respiratory protective devices (RPD). We asked the workers about this information to see how many of the workers use of RPD. The use of RPD during work might reduce respiratory health problems. The questionnaire also included questions about chronic respiratory symptoms—coughing, coughing with sputum, breathlessness, work related shortness of breath, wheezing, and chronic bronchitis. 

#### 2.5.2. Lung Function Test

Lung function tests were performed for a total of 225 participants (i.e., 115 coffee workers and 110 control workers), according to the ATS [17] recommendation for spirometry. A portable spirometer (SPIRARE 3 sensor model SPS 320) was used to measure the lung function. The standing height and weight of the participants was measured using standard weight and height measure. The test was performed during the day shift between 08:00 and 16:00, with the workers in a sitting position. Three acceptable maneuvers with consistent (“repeatable”) results were retained and the best of all of these was recorded. Only the absolute values for the lung function are given in the results, as there are currently no reference equations for the Ethiopia population for obtaining predicted values. The lung function parameters included were FVC and FEV_1_, as well as the percentage ratio of FEV_1_/FVC. The participants with FEV_1_/FVC < 0.70 were considered to have airflow limitations [18]. FEV1 is the maximal volume of air exhaled in the first second of a forced expiration from a position of full inspiration. FVC is the maximal volume of air exhaled with maximally forced effort from a maximal inspiration.

Eleven spirometer results among coffee workers and seven among the controls were excluded from the analysis because of unacceptable readings.

### 2.6. Operational Definition of Variables

Current smoker: participants who smoke currently or those who stopped smoking less than one year ago.

Ex-smoker: participants who had stopped smoking more than one year ago.

Never smoker: participants who had never smoked.

Cough: participants were considered to have coughed if they answered “yes” to at least one of the following four questions; cough first thing in the morning, cough during the day or night, cough as much as four to six times a day in a week, or cough for most days for as much as three consecutive months during the year.

Cough with sputum: participants were considered to have cough with sputum if they answered “yes” to at least one of the four questions: cough with sputum first thing in the morning, cough with sputum during the day or night, cough with sputum as much as four to six times a day in a week, or cough with sputum for most days for as much as three consecutive months during the year.

Breathlessness: participants were considered to have breathlessness if he/she was troubled by a shortness of breath when hurrying on level ground or walking up a slight hill, or get shortness of breath when walking at his/her own pace on the level ground.

Work-related shortness of breath participants were considered to have work-related shortness of breath if he/she usually experience chest tightness while at work or just after work.

Wheezing: participants were considered to have wheezing if his/her chest ever sounded wheezy (whistling sound).

### 2.7. Data Management and Analysis

The collected data were checked for completeness and consistencies by the principal investigator through a close follow up during the data collection period. The data were coded, and no names were included in the database. The code list as well as the data were kept confidential, and were accessed only by the research team.

Independent *t*-tests were used to compare mean values for the continuous variables. The Pearson chi-square test or Fisher’s exact test, if the expected value was less than 5, were used to test the difference between the groups regarding the categorical variables. Poisson regression analysis with a robust variance was used to determine the prevalence ratio of the different respiratory symptoms between the coffee workers and controls, with a corresponding 95% confidence interval, and the statistical significance level was set to a *p*-value less than 0.05. As the prevalence of the chronic respiratory symptoms was high, we chose the prevalence ratio over the odds ratio, because the odds ratio overestimated the strength of association [19].

Analysis of covariance (ANCOVA) were used to compare the mean lung function parameters between the coffee workers and controls when adjusting for height and education level. We adjusted for education level, because there was a significant difference in the educational level between the coffee workers and the controls.

### 2.8. Ethical Approval

The Institutional Review Board of the College of Health Sciences of Addis Ababa University (Protocol number: 051/15/SPH) and the National Research Ethical Review Committee of the Federal Ministry of Science and Technology (NRERC-3/10/110/2016) approved the study. Permission to conduct the study was obtained from the factory managers. Written informed consent was obtained from each participant, and participation in the study was voluntary. Participants with lung function impairments were advised to consult the nearest health center.

## 3. Results

### 3.1. Characteristics of the Study Participants

All of the participants were men. A total of 115 coffee workers and 111 controls participated in the study, making the response rate 94%. The reasons for non-response were that five workers refused to participate, seven workers were in sick leave, and two had stopped working. The coffee workers were older and had a lower educational level than the controls (Table 1). No difference was found between the groups regarding weight, height, BMI, and past respiratory diseases (Table 1).

### 3.2. Use of Respiratory Protective Device (RPD)

The majority of the 112 (97.4%) coffee workers did not use any type of respiratory protective devices (RPD). Among the non-users of RPD, 109 (97.3%) of the coffee workers indicated that the reason for not using RPD was because it was not available or not provided at the work place. Others reported that the reasons for not using RPD were because it was not comfortable (one worker) (0.9%), and that the worker experienced that the RPD did not protect from the dust (one worker) (0.9%).

### 3.3. Chronic Respiratory Symptoms

The prevalence of chronic respiratory symptoms was in the range of 5.2–55% and 2.7–12.7% among the coffee workers and controls respectively (Table 2). Six of the coffee workers (5.2%), and none of the controls had chronic bronchitis. The prevalence ratio of all of the respiratory symptoms was significantly higher for the coffee workers compared to the controls after adjusting for age, education, years worked in other dusty factories, and previous respiratory disease (Table 2). As the number of current smokers were few, the analysis was also performed after excluding the smokers. This did not change the results substantially.

### 3.4. Lung Function

Table 3 shows the result of lung function stratified age among the coffee workers and controls. The coffee workers in the age group 28–39 years and ≥40 years, had significantly lower FVC and FEV_1_ compared with the controls in the similar age category. The FEV_1_/FVC-ratio was significantly lower among the coffee workers compared to the controls in the oldest age group (Table 3). The prevalence of airflow limitation (FEV_1_/FVC < 0.7) was higher among the coffee workers compared to the controls in all of the age categories (Table 3). The analyses were also performed again without including education level in the model, and the results were quite similar (data not shown).

## 4. Discussion

This study found a significantly higher prevalence of respiratory symptoms and lower lung function among the coffee workers compared to controls. Our results are consistent with studies conducted in primary coffee processing factories in Papua New Guinea, Uganda, and Tanzania [8,9,11]. All of these studies show that coffee workers have high prevalence of respiratory health problems. However, our present study found a higher prevalence of some of the respiratory symptoms compared with the studies conducted among Arabica coffee workers in Tanzania, where the prevalence of breathlessness was 14%, wheezing 13%, and chronic bronchitis 3.1% [12]. One of the reasons for this difference could be the higher personal total dust exposure in Ethiopian coffee factories (GM = 12.3 mg/m^3^) compared with the comparable job groups in Tanzanian primary coffee factories (GM = 2.1 mg/m^3^) [20]. The different methods of coffee pre-processing could be another reason; the Arabica coffee is pre-processed only by a wet-method in the Tanzanian factories, whereas in Ethiopia, Arabica coffee is pre-processed either by dry or wet method, based on the individual farmers’ interests. In addition, only 3% of the workers used RPD in Ethiopia, compared to 33% in the coffee workers in Tanzania. The lack of RPD use makes it more likely that the workers actually were exposed to the dust levels measured in the factories. There may have been other additional factors that were not identified in the present study that may also have influenced the respiratory health of the workers.

Similarly, the prevalence of a cough with sputum and wheezing in our study was higher than in the study in Uganda; where the prevalence was 5.2% and 13.5%, respectively [9]. This might be due to the different types of coffee species between the two countries; the Robusta and Arabic coffee were processed in Ugandian factories, whereas only Arabica coffee was processed in Ethiopia.

For a cough with sputum, we found a higher prevalence than reported in the study in Papua New Guinea (8.7%) [8]. This difference might be due to higher dust exposure in the present study compared with what was measured in Papua New Guinea (0.7–10 mg/m^3^). In addition, the difference in working environments, coffee processing methods, and level of awareness among the coffee workers about the impact of dust exposure on their health might be the reason for the difference in the symptom prevalence. In addition, there may be differences between these countries regarding the presence of, for instance, lung infections or sequela after lung infections. Infections may cause respiratory symptoms and influence lung function. This possibility is not very likely, as the examined workers are performing hard physical work, but this factor needs to be considered, because of the high prevalence of tuberculosis as well as HIV in East-African countries [21]. However, it is not likely that this type of health problem is different among the workers in the two factory types included in our study.

The present study’s results showed that in the two oldest age groups of coffee workers, the FVC and FEV_1_ were lower than among the controls, while FEV_1_/FVC was lower in the oldest age group of coffee workers than among the controls. In the Tanzanian study, there were no difference in the FVC and FEV_1_ between coffee workers and controls [12]. The considerably higher dust exposure in the Ethiopian study (GM = 12.3 mg/m^3^) [13] compared to the levels reported for the processing of Arabica coffee in Tanzania (GM = 2.1 mg/m^3^) [20] may have contributed to the difference in findings related to lung function. Also, the study conducted in Papua New Guinea, where the dust levels were lower than in the present study, did not find significant differences in FVC and FEV_1_/FVC between the coffee workers and controls.

Both FEV_1_ and FVC were reduced among the coffee workers, indicating both obstructive and restrictive lung effects. However, in the oldest age group, the FEV_1_/FVC ratio was less than 0.70 for about 27% of the coffee workers, which indicates the presence of an obstructive lung disease [18]. It is noteworthy that such a result was found, even though these workers were present at the workplaces in physically demanding work.

No statistically significant difference was observed in the incidence of past respiratory diseases between the coffee workers and controls; unfortunately, we have no information about when these past respiratory diseases occurred. For example, they could have been before starting work in coffee factories or after starting working in the factories. We only asked the participants if they had ever had any previous respiratory disease.

The control group in the present study was from another production factory, with very low dust levels. Another possibility would have been to choose a control group from the general population, but this would have introduced other types of bias in the study, related to socioeconomic differences between the factory workers and the population.

This is the first study to assess the prevalence respiratory symptoms and lung function among coffee workers in Ethiopia. However, as this study is a cross sectional study, the cause and effect association between dust exposure, and respiratory symptoms and lung function reduction cannot be drawn based on our findings. Our analyses were adjusted for other factors, including age, which may affect lung function. However, it is worth noting that there may be other variables present, which we have not identified. We would therefore recommend that a longitudinal study is undertaken in order to characterize the association between dust exposure and lung function reduction.

This study used a questionnaire-based interview to assess the respiratory symptoms that might result in recall and interviewer bias. However, similar questions were used to assess the respiratory symptoms in both the coffee workers and control groups. To minimize bias, a well-trained interviewer was involved in interviewing both groups. Also, the reported symptoms agreed with the objective measures from the spirometry. Symptoms such as coughing, wheezing, and breathlessness are often associated with obstructive lung disease [22].

This study included workers from all three coffee growing and processing regions of Ethiopia, and the factories are considered to be representative to all similar primary coffee processing factories in Ethiopia. Similar results might also be expected by any African coffee factory with a similar dust exposure level and similar production type of Arabica coffee beans.

As this study showed that the majority of the coffee workers did not use any type of respiratory protective devices, an immediate action to reduce respiratory health problems among coffee workers would be to provide proper facemasks.

## 5. Conclusions

Workers in primary coffee processing factories in Ethiopia had a higher prevalence of chronic respiratory symptoms and lower lung function than the controls. This might represent early signs of lung disease. A longitudinal study on the possible effects of coffee dust on respiratory health among coffee production workers is recommended.

## Figures and Tables

**Table 1:** Characteristics of the participants.

Variable	Coffee Workers (n = 115)	Control (n = 110)	*p*-Value
Age (years); AM (range)	35.1 (18.0–75.0)	30.9 (18.0–71.0)	0.008 ^$^
Weight (Kg); AM (range)	59.8 (43.0–88.0)	60.0 (45.0–90.0)	0.9 ^$^
Height (cm); AM (range)	169.3 (148.0–187.0)	169.9 (153.0–183.0)	0.5 ^$^
BMI (Kg/m^2^); AM (range)	20.8 (15.4–30.5)	20.7 (16.6–29.1)	0.8 ^$^
Duration of employment at present work years AM (range)	6.5 (1.0–30.0)	3.4 (1.0–6.0)	0.001 ^$^
Years worked in other dusty factories AM (range)	0.23 (0.0–12.0)	0.5 (0.0–12.0)	0.2 ^$^
**Education**			
Illiterate; n (%)	14 (12.2)	3 (2.7)	0.007 ^†^
Primary education & above; n (%)	101 (87.8)	107 (97.3)	0.007 ^†^
**Smoking habits**			
Ex smoker; n (%)	4 (3.5)	1 (0.9)	0.37 ^±^
Current smoker; n (%)	3 (2.6)	4 (3.6)	0.71 ^±^
Cigarettes smoked per day for current smokers AM (range)	3 (2–4)	2 (1–3)	0.2 ^$^
**Cooking**			
Cooking food at home; n (%)	103 (89.6)	97 (82.2)	0.74 ^†^
Kitchen located inside the living room; n (%)	34 (33)	31 (32)	0.87 ^†^
Use biomass for cooking; n (%)	96 (93.2)	86 (88.7)	0.26 ^†^
**Previous Respiratory Disease**			
Pneumonia; n (%)	7 (6.1)	5 (4.5)	0.61 ^†^
Bronchitis; n (%)	3 (2.6)	1 (0.9)	0.62 ^±^
Tuberculosis; n (%)	4 (3.5)	2 (1.8)	0.68 ^±^
Asthma; n (%)	8 (7)	4 (3.6)	0.27 ^±^
Participants who have had at least one of the respiratory diseases, n (%)	22 (19.1)	12 (10.9)	0.09 ^†^

AM: arithmetic mean; ^$^ Independent *t* test between control and coffee workers; ^†^ Pearson chi square test; ^±^ Fisher’s exact test. BMI—body mass index; “n”: Number of study participants.

**Table 2:** Prevalence of chronic respiratory symptoms among coffee workers and controls.

Variable	Coffee Workers n = 115	Control n = 110	Prevalence Ratio, 95% CI	*p*-Value
Cough; n (%)	52 (46.4)	8 (7.5)	5.6 (2.9–11.7)	<0.001
Cough with sputum; n (%)	26 (23.2)	2 (1.9)	11.4 (2.7–47.8)	<0.001
Breathlessness; n (%)	40 (35.7)	12 (11.3)	2.9 (1.6–5.3)	<0.001
Work-related shortness of breath; n (%)	22 (19.6)	6 (5.5)	3.7 (1.6–8.7)	0.003
Wheezing; n (%)	20 (17.9)	5 (4.7)	3.3 (1.3–8.4)	0.01

CI, confidence interval while adjusting for age; education, years worked in other dusty factories and previous respiratory disease; *p*-value when comparing coffee workers vs. controls; “n”: Number of study participants.

**Table 3:** Lung function among primary coffee processing factories and control groups stratified by age (three age groups).

Lung Function Parameters	Age Group	No of Participants		Absolute Value Mean (SD)		*p*-Value
		Coffee Workers n = 104	Controls n = 103	Coffee Workers	Controls	
FVC (l)	18–27	35	57	4.60 (0.43)	4.70 (0.49)	0.35
	28–39	35	23	4.08 (0.47)	4.43 (0.46)	<0.001
	≥40	34	23	3.38 (0.53)	3.67 (0.51)	0.05
	All age group	104	103	4.03 (0.69)	4.41 (0.63)	<0.001
FEV_1_ (l)	18–27	35	57	3.74 (0.46)	3.87 (0.39)	0.15
	28–39	35	23	3.29 (0.58)	3.63 (0.47)	0.02
	≥40	34	23	2.60 (0.50)	3.05 (0.51)	<0.001
	All age group	104	103	3.22 (0.69)	3.63 (0.54)	<0.001
FEV_1_/FVC	18–27	35	57	0.81 (0.08)	0.83 (0.05)	0.43
	28–39	35	23	0.80 (0.09)	0.82 (0.07)	0.48
	≥40	34	23	0.77 (0.09)	0.83 (0.06)	0.02
	All age group	104	103	0.80 (0.09)	0.83 (0.06)	0.01
FEV_1_/FVC < 0.7; n (%)	18–27	35	57	3 (8.6)	0	-
	28–39	35	23	2 (5.7)	1 (4.3)	1 *
	≥40	34	23	9 (26.5)	0	-

Analysis of covariance between coffee worker and controls while adjusting for height and education level. * Fisher exact test between coffee workers and controls; *p*-value: significance level; “n”: Number of study participants.
